# Adipocyte Microenvironment in Ovarian Cancer: A Critical Contributor?

**DOI:** 10.3390/ijms242316589

**Published:** 2023-11-22

**Authors:** Ana Duarte Mendes, Ana Rita Freitas, Rodrigo Vicente, Marina Vitorino, Marta Vaz Batista, Michelle Silva, Sofia Braga

**Affiliations:** 1 Medical Oncology Department, Hospital Prof. Doutor Fernando Fonseca, 2720-276 Amadora, Portugal; rita.freitas@hff.min-saude.pt (A.R.F.); rodrigo.vicente@hff.min-saude.pt (R.V.); marina.vitorino@hff.min-saude.pt (M.V.); marta.vaz@hff.min-saude.pt (M.V.B.); michelle.c.silva@hff.min-saude.pt (M.S.); sofia.braga@hff.min-saude.pt (S.B.); 2 Haematology and Oncology Department, CUF Oncology 2710-204 Sintra, Portugal; 3 Haematology and Oncology Department, CUF Oncology, 1998-018 Lisbon, Portugal

**Keywords:** tumor microenvironment, ovarian cancer, adipocyte, pathogenesis, peritoneal metastasis

## Abstract

Ovarian cancer is one of the most common gynecological malignancies and has low survival rates. One of the main determinants of this unfavorable prognosis is the high rate of peritoneal metastasis at diagnosis, closely related to its morbidity and mortality. The mechanism underlying peritoneal carcinomatosis is not clearly defined, but a clear preference for omental spread has been described. Growing evidence suggests that adipose tissue plays a role in promoting cancer onset and progression. Moreover, obesity can lead to changes in the original functions of adipocytes, resulting in metabolic and inflammatory changes in the adipose tissue microenvironment, potentially increasing the risk of tumor growth. However, the specific roles of adipocytes in ovarian cancer have not yet been fully elucidated. Due to the undeniable link between obesity and cancer, the adipose tissue microenvironment could also present a promising therapeutic target that warrants further research. This review discusses the complex relationship between ovarian cancer and the adipose tissue microenvironment.

## 1. Introduction

Ovarian cancer (OC) ranks as the eighth most prevalent cancer and the fifth deadliest cancer among women globally. Its occurrence is 3.4%, while mortality is 4.7%, resulting in around 200,000 deaths per year due to OC. This highlights the significant disease burden on women’s health and survival [1]. Since OC tends to advance rapidly and exhibits no apparent signs or symptoms in its early stages, more than two-thirds of patients are diagnosed at an advanced stage [2].

Despite extensive surgical cytoreduction and newer adjuvant treatments, the survival rates for stage III and stage IV remain disappointingly low at 40% and 20%, respectively [3]. Within two years, more than half of the patients will experience a relapse, which ultimately results in a detrimental effect on survival rates [4].

Peritoneal metastasis is a common occurrence in patients with OC [5]. It contributes to malignant ascites accumulation due to the complex tumor-promoting microenvironmental network in the peritoneal cavity. Although the peritoneal cavity is a frequently affected site for OC metastasis, there is still a lack of understanding of the mechanisms related to the proliferation of metastases in the intra-abdominal environment. OC seems to prefer spreading to the adipose tissue (AT) within the abdominal area, specifically the omentum. By further investigating the potential mechanisms involved, we may enhance molecular diagnosis and treatment options [6]. The intricacies of the tumor microenvironment (TME) within the peritoneal cavity and intra-abdominal metastasis remain poorly understood. Comprised of various cell types, including tumor and host stromal cells, blood vessels, and the extracellular matrix (ECM), the TME’s role in OC cell dissemination is yet uncertain. Nevertheless, research suggests that the interaction between tumor and stromal cells may facilitate OC cell spread within the peritoneal cavity [7,8].

Studies have shown that adipocytes, a vital component of the stromal microenvironment in multiple cancers, have a tumor-promoting effect on various tumor cells at a molecular level. Cancer-associated adipocytes are especially significant in cancer progression since they facilitate cell growth, angiogenesis, anti-apoptotic effects, and migration directly or indirectly [9]. Furthermore, certain adipokines, such as leptin, interleukin (IL) 6, and IL-8, have been identified to contribute to the growth, spread, and drug resistance of various types of tumors. These findings suggest that an environment rich in adipocytes may promote tumor development [10].

Obesity, which refers to excessive AT growth, is an increasingly pressing global issue [11]. Given the staggering prevalence of obesity globally, it is no wonder it has become nearly as significant as tobacco use, a preventable risk factor for cancer. Research indicates that it contributes to roughly 14% of cancer-related fatalities in males and approximately 20% in females [12]. Being overweight or obese can increase the risk of various types of cancer, including colorectal cancer, postmenopausal breast cancer, endometrial cancer, gall bladder cancer, pancreatic cancer, kidney cancer, OC, and liver cancer [13]. It should be noted, however, that a higher BMI is associated with a lower risk of breast cancer in premenopausal women [14].

In addition to the indisputable epidemiological data, the molecular effects of obesity have been a topic of significant discussion. Obesity plays an important role in the molecular modulation of normal AT: recent evidence suggests that obesity can cause adipocytes to become proinflammatory, dysfunctional, and senescent-like [15]. Although the connection between OC and adipocytes remains incompletely understood, further research is being conducted to elucidate the functional links between obesity and cancer at the molecular and cellular levels.

In this review, we will explore the possible correlation between an adipocyte-rich TME and OC.

## 2. Ovarian Cancer

Ovarian cancer is a serious health concern affecting thousands of patients worldwide. It is estimated that approximately 23% of gynecologic cancers are ovarian, and women with OC account for 47% of all deaths from cancer of the female genital tract. The incidence rates of OC vary geographically, ranging from 9 to 17 per 100,000 people. The highest rates are observed in high-income countries, except for Japan. The incidence is known to increase with age, with most patients falling under the 60–64 age group. In low-income countries, the median age at diagnosis typically occurs a decade earlier [1,16].

Research has provided insights into identifying different subtypes of OC, emphasizing their diversity. These tumors exhibit various histological and genomic features, thus having distinct prognoses and chemosensitivity. Despite this improved knowledge, we have seen few therapeutic advances [17]. OC can be classified into two different histological subtypes: epithelial OC (EOC) and non-epithelial OC, each with various subtypes. EOC is the most common type, comprising approximately 90% of all ovarian malignancies. Molecular and genetic studies have also improved our understanding of OC development. Two main categories of ovarian carcinogenesis based on a molecular classification have been identified: type I and type II. Type I tumors have well-defined precursor lesions and exhibit a slower growth rate. These tumors are typically identified in the early stages of development and have a stable genome, although somatic mutations are frequently detected. Type II tumors, on the other hand, are more aggressive. They are usually detected at an advanced stage and exhibit high genetic instability. At the molecular level, type I tumors are characterized by a stable genome without tumor protein 53 (*TP53*) mutations. However, type II tumors show *TP53* mutations in nearly 80% of cases and a high Ki67 proliferation index (between 50% and 75%). They also present an increased incidence of breast cancer gene (*BRCA*) 1/2 [18]. Regarding histological origins, type I tumors appear to form part of a morphological continuum, starting from benign extraovarian tumors, and afterward confined to the ovary. On the other hand, recent evidence suggests that type II tumors are not linked to precursor lesions in the ovary and originate primarily from the epithelium of the fimbrial portion of the fallopian tube [19].

Based on the 2020 classification by the World Health Organization (WHO) regarding tissue samples, immunohistochemistry, and molecular analysis, there are at least five subtypes of malignant EOC. These are the high-grade serous carcinoma (HGSOC), which accounts for approximately 70% of cases, endometrioid carcinoma (EC) in about 10%, clear-cell carcinoma (up to 10%), low-grade serous carcinoma (LGSC) that can reach 5% of all ovarian malignancies and mucinous carcinoma in about 3–4% of them. Other rare types include mixed-cell tumors, mesonephric-like carcinoma, malignant Brenner tumors, carcinosarcoma, and undifferentiated carcinoma. Less frequently encountered are the non-epithelial tumors, which typically manifest in younger women. They encompass germline tumors, sex cord-stromal tumors, and various uncommon types like small cell cancers [17,18,19,20,21].

EOC is frequently linked to mutations in four essential genes: *TP53*, *BRCA1/2*, phosphatidylinositol-4,5-bisphosphate 3-kinase catalytic subunit alpha (*PIK3CA*), and Kirsten rat sarcoma virus (*KRAS*).

Mutations in the *BCRA1* and *BCRA2* genes are among the most relevant genetic risk factors. Germline *BRCA1* mutations are the most common hereditary factor associated with HGSOC, and approximately 50% of cases also exhibit *P53* mutations. Meanwhile, *PIK3CA* mutations are more often associated with clear-cell carcinoma and endometrioid OC, while *KRAS* mutation plays a crucial role in LGSC and mucinous OC. Recent evidence suggests that many OC patients exhibit gene alterations responsible for DNA repair and homologous recombination [18,22,23].

Most OC cases are sporadic, with only 10% attributed to genetic predisposition. Most of these are due to pathogenic *BRCA* 1 or 2 gene mutations. It is imperative that genetic testing be offered to all women who are suspected of carrying a *BRCA* gene mutation, mainly those with a family history, young age of diagnosis, or high-grade serous or endometrioid cancer [21,22,23]. In addition to *BRCA*, it is also essential to mention Lynch syndrome (LS) since the cumulative risk of OC among carriers varies between 6–12%. This syndrome is an autosomal dominant condition with a high predisposition to develop cancer. It is characterized by germline mutations in one of four DNA mismatch repair (MMR) genes: *MLH1*, *MSH2*, *MSH6*, and *PMS2*. OC in women with LS presents distinct characteristics in comparison to sporadic OC and OC in *BRCA* 1/2 mutation carriers. Specifically, it tends to manifest at a younger age, exhibit a non-serous histological type, and be diagnosed earlier. These clinical characteristics could frame the OC associated with LS as a Type 1 EOC. These unique traits have been shown to contribute to a more favorable overall survival rate for those who develop OC [24]. Noteworthy, LS-OC is highly immunogenic, presenting as microsatellite-high and with high levels of CD8+ lymphocites [25].

Several risk factors for OC have been described, such as nulliparity, endometriosis, obesity, and smoking (in mucinous carcinoma). The risk is more prominent among postmenopausal women, with age being a contributing aspect. Conversely, increased parity, first pregnancy at an earlier age, and use of oral contraceptive pills are found to have a protective role [26]. Regarding obesity, a pooled analysis from the Ovarian Cancer Association Consortium (2013) revealed that obesity or overweight is linked to an overall increased risk of OC. Notably, this association seems primarily relevant to non–serous and low–grade serous subtypes when considering invasive cancers [27]. However, a systematic review and meta-analysis by B. Ellwanger et al. concluded a positive association between adiposity and OC among premenopausal women but not in postmenopausal women. Furthermore, this association occurs among cases of mucinous and clear-cell but not serous or endometrioid histology [28]. 

The precise influence of obesity or weight gain on the pathogenesis of *BRCA*-associated OC remains uncertain. Nevertheless, a recent study by Bhardwaj et al. has shed some light by demonstrating a positive correlation between body-mass index and epithelial cell DNA damage. The authors identified additional obesity-associated factors, including leptin and insulin, that increased DNA damage in human *BRCA* heterozygous epithelial cells. Regarding the connection between *BRCA* mutations and OC, Bhardwaj et al. reported increased DNA damage in epithelial cells of the fallopian tubes in *BRCA* carriers who underwent oophorectomy. Surprisingly, no such increase was observed in the ovaries of mutated *BRCA* undergoing oophorectomy [29,30].

Obesity can potentially influence the effectiveness of therapy, with some HGSOC patients who express upregulated genes related to obesity and lipid metabolism showing a poorer outcome [31].

Concerning patterns of disease, nearly 70% of OC patients have peritoneal cavity metastasis at diagnosis, and 75% of recurrent disease is intraperitoneal. Compared to other types of tumors, OC often spreads to the omentum or peritoneum. This is presumably due to the absence of anatomical barriers protecting the ovary epithelium or fallopian tube within the abdominal cavity, allowing cancer cells to detach from the primary tumor easily. Although these cells can disseminate throughout the peritoneal cavity, they are more likely to metastasize to the peritoneum and omentum, where they can flourish in a favorable environment for survival, engraftment, and development through various interactions with stromal cells. Unfortunately, treating intra-abdominal lesions in OC remains challenging, and the added value of intraperitoneal and hyperthermic intraperitoneal chemotherapy is still uncertain [32,33].

## 3. Peritoneal Microenvironment and OC Peritoneal Metastasis

**Figure 1:**
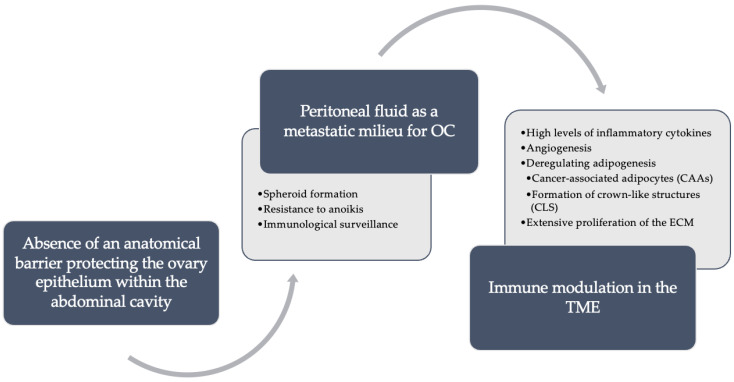
Main pathways regarding peritoneal metastasis. In ovarian cancer (OC), three steps are described as fundamental to the establishment of peritoneal metastasis. The first is an anatomical feature, the fact that there is an absence of an anatomical barrier protecting the ovary epithelium within the abdominal cavity. The second is that peritoneal fluid serves as a metastatic milieu for cell propagation through immune surveillance, resistance to anoikis, and the formation of spheroids. Finally, the modulation of the immune system in the tumor microenvironment (TME). The TME directly modulates the immune system in its favor through four steps: release of inflammatory cytokines, angiogenesis, the extensive and unregulated proliferation of extracellular matrix (ECM), and finally, angiogenesis to the benefit of neoplastic cells.

The mechanisms that enable OC to easily spread to the peritoneum through tumor-stromal cell interaction in the TME are not well understood (Figure 1).

The peritoneal cavity holds serous exudate, which includes steroid hormones, cytokines, and growth factors. The amount of peritoneal fluid in humans can range from 5 to 20 mL and may vary based on the individual’s physiological state [34]. Notably, macrophages are the most abundant immune cell population in peritoneal fluid, followed by smaller populations of T, dendritic, mast, NK, and B cells [35].

The peritoneal fluid provides a metastatic milieu for OC. The microenvironment of ascites is particularly conducive to this process, allowing these cells to disseminate and seed within the peritoneal cavity. Even when detached from the primary tumor, these cancer cells can persist within the ascites by overcoming various obstacles, including spheroid formation, resistance to anoikis, and immunological surveillance [8,36].

The deregulation of the expression of surface proteins, such as integrin and fibronectin, facilitates the aggregation of tumor cells. In vitro studies have shown that the upregulation of integrin α5 in tumor cells favors the formation of aggregates between these cells and fibroblasts, contributing to the early stages of peritoneal dissemination. These mechanisms are yet to be fully understood [37].

After detachment from the primary sites, OC cells acquire anoikis resistance to survive within the peritoneal cavity. This process of resistance is related to the ability of tumor cells to overcome the process of apoptosis after losing connections with the cellular matrix of the primary site [38]. Several molecular processes are involved in this mechanism of tumor cell resistance, namely Ras-related protein Rab-25 (RAB25) small GTPase, integrin members, and leucine rich repeat containing 15 (LRRC15) [35].

In the TME, immune modulation by tumor cells in the peritoneal cavity is one of the most important mechanisms for survival and proliferation. In peritoneal fluid, the detection of high levels of inflammatory cytokines such as IL-2 and TNF are indicators of tumor cell activity [39]. Besides, OC cells in ascites induce apoptosis of CD95-positive immune cells and could also recruit regulatory T cells (Treg) and inhibit specific anti-tumor immunity, promoting tumor progression [40].

The omentum is the fused peritoneal fold nestling on the surface of the intra-peritoneal Worgans. It is a critical component in immune regulation by managing inflammation, fluid exchange, promoting angiogenesis, and regulating adipogenesis. Beneath its mesothelial cell covering lays adipocytes, adipose-derived stromal cells, fibroblasts, and immune cells [41]. Milky spots are lymphoid structures found in the omentum, which consists mainly of macrophages, T cells, and B cells. It is worth noting that the mesothelial lining in these spots is not continuous, which allows leukocytes to relocate into the peritoneal cavity. 

**Figure 2:**
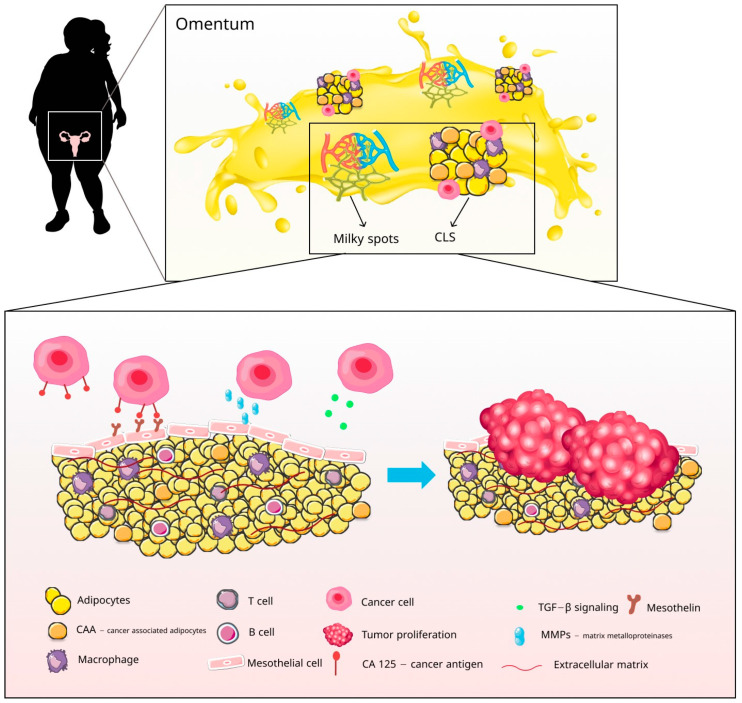
Interaction between ovarian cancer (OC) cells and adipose tissue (AT) within the tumor microenvironment (TME). The microenvironment within the peritoneal cavity is crucial for OC pathogenesis. The close collaboration between cancer cells, stromal cells, and adipocytes produces outcomes that flow in both directions. The stroma and AT play a vital role as a pre-metastatic niche, while cancer cells disrupt the normal adipose and stromal tissue, adapting it to their needs and creating an environment more conducive to cancer growth. Pro-inflammatory macrophages form a ring around dying and necrotic adipocytes (CAA—cancer-associated adipocytes), forming a crown-like structure (CLS). The development of cancer cells in the peritoneum tends to occur in the omental milky spots, lymphoid structures found in the omentum. CA125 and the overexpression of MMPs promote the adhesion of cancer cells into the peritoneal mesothelial layer. Afterward, the close interaction between CAA in the omentum and cancer cells stimulates tumor proliferation by various mechanisms: boosting a vast extracellular matrix (ECM), inducing a senescent-like phenotype, exchanging metabolites, and promoting immune regulation.

Various cells play an essential role in shaping the TME of the peritoneal cavity, namely tumor cells, stromal cells, adipocytes, and immune system cells. The preparation of the pre-metastatic niche, a crucial component for the spread of OC, is facilitated by the interaction between tumor cells and stromal cells in the omentum [42] (Figure 2). 

The tumor cells are of utmost significance among these cells, as they create pre-metastatic niches and trigger a mesothelial-to-mesenchymal transition (MMT). Ordinarily, mesothelial cells serve to prevent cancer cell adhesion to the peritoneum. However, when MMT occurs, they foster adhesion and metastasis. Fibroblasts serve as primary ECM regulators, but they can also promote metastasis by transforming into cancer-associated fibroblasts (CAFs). Although the precise mechanisms behind this transformation are not yet fully understood, the impact of CAFs is unmistakable. They facilitate peritoneal metastasis by regulating the ECM, secreting cytokines, TGF-β, and vascular endothelial growth factor (VEGF) [43]. The extensive proliferation of the ECM in peritoneal carcinomatosis associated with OC poses a significant challenge for systemic therapy penetration. Tumor cells can induce MMT of peritoneal mesothelial cells, resulting in further ECM proliferation [44]. Studies have revealed that OC cells manipulate the TME by regulating the ECM, facilitating a favorable tumor growth environment [45]. This regulation can be conducted by an aberrant transforming growth factor beta (TGF-β) pathway. TGF-β can be released from tumor cells, contributing to regulating epithelial-to-mesenchymal (EMT) transition and metastatic niche formation. Studies have shown that high-grade OC exhibits a high level of amplification in the TGFβ pathway. Research suggests that all three isoforms are elevated in primary, metastatic, and recurrent EOCs compared to normal ovaries. The fallopian tube, one of the sites of tumor initiation and early metastasis of high-grade serous OC, expresses the TGFβ isoforms and their receptors. Interestingly, while tumor cells are a source of TGFβ in OC, the peritoneal mesothelium and tumor-infiltrating cells also contribute to its production. Tumor cells in the peritoneal environment have been found to interact with mesothelial cells through TGFβ signaling. This signaling pathway is mediated by cancer-derived TGFβ1. As a result, the expression of fibronectin is increased in peritoneal mesothelial cells [46].

Cancer antigen 125 (CA125) is a serum tumor marker commonly used in clinical practice. It is a glycoprotein overexpressed on OC cells that promotes the adhesion of OC cells into the peritoneal mesothelial layer through binding to mesothelin [47]. 

The basement membrane of peritoneal tissues is rich in collagen and fibronectin. The disseminated OC cells’ overexpression of matrix metalloproteinases (MMP), like MMP2, facilitates the connection to peritoneal mesothelium through cleaving fibronectin and vitronectin [44]. In addition, OC cells can attach to the peritoneal mesothelium by regulating hyaluronan expression on mesothelial cells.

The immune system also plays a pivotal role in the pathogenesis of OC. Infiltration of regulatory T-cells (Tregs), a subset of T-cells, has been associated with worse outcomes due to impaired anti-tumor immune response. Tregs release TGF-β, which supports the TME [43]. Macrophages in the peritoneal cavity also play an essential role. In normal circumstances, various macrophage populations are present in the peritoneal fluid and peritoneum, each with unique traits [36]. However, tumor-associated macrophages have distinct characteristics and are believed to promote tumor advancement [48,49].

Macrophage-derived cytokines, such as TGF-β1 and macrophage inflammatory protein one beta (MIP-1β), promote cancer cell adhesion, invasion, and proliferation [50].

Recent studies reveal that tumor cells can thrive on nutrients from adipocytes found in peritoneal metastases [51].

The TME is widely recognized as a key factor in the development and progression of tumors throughout the body. In fact, several therapies targeting the TME have been extensively described [52]. This complex network includes a variety of cells, including lymphocytes, antigen-presenting cells, cancer fibroblasts, and the ECM. When an individual becomes overweight, chronic inflammation and hypoxia of the AT can negatively impact this environment, leading to damage to its intricate connections and an increased risk of cancer [53].

## 4. Obesity and Adipogenesis

Adipose tissue, the primary component of the human body, is a loose connective tissue typically situated beneath the skin. However, it can also be deposited in muscles, intestines, omentum, and bone marrow. The functional classification of AT distinguishes between energy-storing white adipocytes, the predominant cell type of white AT (WAT), and thermogenically active brown adipocytes in brown AT, which have distinct cellular organizations and different biological and physiological functions [54]. The location within the human body also significantly alters its function: the accumulation of visceral WAT, particularly in the omentum and mesentery, during weight gain, is strongly correlated with the development of insulin resistance and metabolic syndrome, as opposed to subcutaneous WAT accumulation [55].

Apart from the adipocyte itself, the AT niche is a complex biological entity made up of various cells, including endothelial cells, macrophages, immune cells, and stem cells within adipose stromal cells. Together, these cells work in harmony with secreted factors and ECM to regulate AT homeostasis and remodeling [42].

Initially, adipocytes, the core elements of AT, were believed to function only as storage units for energy. In 1994, AT was recognized as a secreting organ for producing the hormone leptin [56], and over 400 additional factors secreted by adipocytes, known as adipokines, have been identified. These adipokines include hormones like adiponectin, leptin, and resistin, as well as inflammatory cytokines like tumor necrosis factor-α (TNF-α), interleukin (IL)-6, and IL-8, enzymes like 17β-hydroxysteroid dehydrogenase (17βHSD) and 11βHSD1, and other factors. Unfortunately, some adipokines, such as IL-6, IL-8, and leptin, have been found to hasten certain tumors’ growth, metastasis, and drug resistance [57,58].

Obesity is a pathological condition that results in excessive AT growth. It is a widespread problem that is linked to all causes-morbidity and mortality rates, with growing evidence linking obesity to cancer. The World Health Organization has classified it as a non-infectious and non-communicable pandemic [31]. Regarding our field of work, a global study suggested that young women between the ages of 15–40 in certain countries are more susceptible to OC due to elevated obesity and metabolic syndrome rates [59]. Numerous studies utilize plain indicators of obesity, such as body mass index (BMI) or waist circumference, which do not consider the intricate biology involved in being overweight or obese. Although the quantity of fat tissue in the body is associated with an increased risk of disease, it is also crucial to consider the quality of that tissue, including elements like inflammation, adipocyte hypertrophy, and hypoxia [60].

In the process of weight gain, lipids accumulate in the adipocytes, becoming hypertrophic and ultimately dying. The death of these cells triggers our immune system, accumulating immune cells in the area and can modulate the microenvironment to a state of chronic low-grade inflammation. The inflammation has mainly been attributed to areas of AT where infiltrating macrophages surround the dying adipocytes to form crown-like structures (CLS). These structures, existing in most AT, show greater frequency in visceral AT than in subcutaneous tissue, and their number increase with body mass index [61,62]. This process can cause changes in the adipokine profile, which includes a decrease in adiponectin and an increase in leptin, TNF-α, and IL-6. These changes can lead to metabolic and inflammatory alterations in the AT, creating dysfunctional adipocytes and, thus, a favorable environment for tumor development, resulting in a worse cancer prognosis [12,63]. The association of CLS with worse outcomes in cancer patients leads to increased interest as a prognostic biomarker [64]. Aside from the CLSs, various granulocytic cells become active within the myeloid compartment. Neutrophils, highly associated with cancer, rapidly increase in WAT after three days of a high-fat diet. This increase triggers the release of the protease neutrophil elastase, which breaks down insulin receptor substrate 1 (IRS1). This prevents IRS1 from binding to phosphoinositide 3-kinase (PI3K), which leads to insulin resistance. This resistance can promote epithelial cell proliferation, which may act as a surrogate mechanism of PI3K activation in wild-type tumors through its inductive effects on insulin. Although granulocyte dynamics initially help control inflammation during weight gain, they may lower the threshold to malignant transformation by impairing immune surveillance [65,66].

In AT growth, the capillary networks become insufficient to match the needs. This effect results in areas of hypoxia that simulate the ones present in developing tumors. Both obesity and cancer are linked to hypoxia, which triggers the production of pro-angiogenic factors like VEGF and hypoxia-inducible factor 1 subunit alpha (HIF-1α). HIF-1 is critical in various aspects of cancer, such as angiogenesis, stem cell maintenance, metabolic reprogramming, metastasis, and resistance to radiation and chemotherapy. However, even with the angiogenic stimulus, hypoxia in obesity cannot be fully reversed, resulting in persistently low oxygen levels due to poorly functioning blood vessels. This chronic condition leads to adipocyte death, promoting macrophage infiltration [67,68].

Beyond the low-grade chronic inflammation as a bridge to oncogenesis, obesity also plays a crucial role in insulin resistance syndrome. This syndrome has been known to promote cancer development by affecting insulin receptors directly or indirectly by impacting other modulators, such as the insulin-like growth factor (IGF) family of receptors. Adipocytes release IGF-I, a vital growth factor that sustains their differentiation and metabolic regulation. However, altered levels of IGF-I may stimulate tumor malignancies. IGF-1 binds to IGF-1R, a tyrosine kinase receptor, which activates downstream signal effectors, including Ras/Raf/ERK and PI3K/Akt/mTOR. These effectors play a crucial role in cell growth, proliferation, and various types of cancers. AT is also involved in synthesizing a broad range of bioactive molecules, among which adipokines, a group of peptides mainly produced by AT, have emerged as a significant link between obesity and cancer. Adiponectin is the most abundant adipokine in plasma, and its secretion is strongly related to the circulating levels of other hormones. Interestingly, an inverse association between adiponectin and fasting plasma insulin has been demonstrated. Low levels of adiponectin have been associated with obesity and insulin resistance. Conversely, several pieces of evidence have shown that leptin, whose circulating levels increase proportionally with fat mass, directly or indirectly affects the biology of several cancers. In summary, obesity has been recognized as a potential promoter of cancer growth through several mechanisms [69].

Interestingly, there is also a role for gut microbiota and obesity. Recent research has highlighted the significance of gut microbiota in the pathogenesis of various diseases. Gut microbiota refers to the diverse microorganisms that inhabit the human gut tract. The correlation between the onset and progression of obesity and gut microbiota is well-established; however, the precise association and underlying mechanisms remain elusive. An insightful review by Cheng et al. analyzed the existing data. It revealed that dysregulated microbiota can contribute to obesity through several channels, including disruption of energy homeostasis, lipid metabolism, and chronic low-grade inflammation [70].

Furthermore, the microbiome may promote inflammation and control immune responses to support OC development. Certain bacteria, such as *F. nucleatum* and enterotoxigenic *B. fragilis*, associated with colon cancer have been found to produce proteins that accelerate cell proliferation, migration, and angiogenesis through host Wnt-β-catenin signaling. Evidence also indicates that intestinal dysbiosis can activate macrophages to increase TNF-α and IL-6 production, driving EMT and ultimately leading to advanced OC [71,72].

## 5. Adipocytes in Ovarian Cancer

Cancer cells tend to develop in the omental milky spots within peritoneal fat depots [73].

OC cells closely interact with adipocytes in the omentum, leading to significant phenotypic alterations in the adipocytes (Figure 1). These altered adipocytes, with different characteristics from those of primary adipocytes, are named cancer-associated adipocytes (CAAs) [10,74]. Unlike typical adipocytes, CAAs possess a smaller size, decreased lipid content, and lower levels of adipocyte differentiation markers. Conversely, they exhibit higher levels of adipokines and inflammatory factors such as leptin, MMP-11, chemokine ligand (CCL) 2, CCL5, and IL-6. 

The current understanding is that CAAs are multifaceted and constantly evolving, having many roles in constructing a tumor-promoting microenvironment [75].

The various roles are linked to crucial areas: creating a vast ECM, inducing a senescent-like phenotype, exchanging high-value and high-energy metabolites, and immune regulation.

The extensive ECM in CAAs may be related to an adipocyte-derived overexpression of collagen VI [76]. Prolonged exposure to OC leads to the accumulation of CAAs with brown/beige differentiation and fibroblastic characteristics, thereby significantly reinforcing tumor development [77].

CAAs can remarkably halt the cell cycle while increasing the expression of genes related to cell cycle arrest and decreasing the expression of genes promoting cell growth. Furthermore, it is to be noted that the transition from normal adipocytes to CAAs may encounter cellular aging due to the activation of multiple oncogenes [78]. Caveolin-1 (Cav-1)- the major scaffold protein of caveolae (crucial membrane microdomains)—which plays a crucial role in membrane transport, lipid composition preservation, and signal transduction within cells, has been suggested as a tumor suppressor and a decrease in its expression can promote tumor growth and metastasis. Autophagy induced by Cav-1 may play a crucial role in the interaction between CAAs and cellular senescence [79].

Stromal adipocytes can also interact with cancer cells by exchanging metabolites and amino acids. Extracellular vesicles (EVs) act as messengers between adipocytes and cancer cells, carrying proteins and substrates involved in fatty acid oxidation (FAO) to tumor cells. In obesity, EVs can transport fatty acids and store lipid droplets in cancer cells. The metabolic disorder of CAAs may be linked to altered immunoregulation, possibly through the promotion of FAO or the intake of immunomodulatory amino acids [80,81].

CAAs are crucial in producing various substances such as adipokines, chemokines, cytokines, and exosomes. These can have a significant impact on tumor growth and treatment efficacy. CAAs release chemicals such as CCL2, CCL5, IL-1β, IL-6, TNF-α, VEGF, and leptin, facilitating tumor spread and evading the immune system. Leptin, in particular, has been shown to influence the immune system, as noted in a review by Naylor et al. [82]. Additionally, CCL2 and CCL5 can attract and modify the behavior of macrophages in AT, known as AT macrophages (ATMs), through the release of exosomes. ATMs have been associated with the production of various inflammatory substances [83], and their accumulation is frequently associated with adverse outcomes and resistance to cytotoxic therapy [84].

Adipocytes in brown AT play an immunosuppressive role by expressing programmed death-ligand 1 (PD-L1). This internal pool of PD-L1 could impact antitumor immunity. In vitro, adipocyte surface PD-L1 could interact with T cell surface PD-1 to debilitate the antitumor role of T cells. In vivo, adipocyte PD-L1 could dampen interferon (IFN)γ production of CD8+ T cells. Furthermore, adipogenesis inhibition selectively decreased the PD-L1 expression in AT and enhanced the antitumor effect of anti-PD-L1 or anti-PD-1 antibodies [85].

Given OC’s unique adipocyte-rich metastatic niche, the adipocytes’ immunosuppressive role could explain the low response to checkpoint blockade immunotherapies [86]. It should be noted, however, that the relationship between obesity and response to PD-1 blockade is not completely understood, as the high efficacy of immune checkpoint inhibitors in obese men with melanoma has been described [87].

The evidence is mounting—adipocytes are actively involved in cancer progression.

## 6. Future Directions

It is becoming increasingly clear that cancer and obesity have a close connection. Obesity can lead to inflammation within adipocytes, which could promote tumor development. Additionally, tumor cells can adulterate normal adipocytes into facilitators of tumor proliferation. A substantial body of evidence supports the relationship between cancer and obesity.

Incorporating TME-targeted therapies in addition to conventional tumor cell-focused treatments is a successful approach to combating cancer [53]. Immunotherapy has generated considerable attention, with numerous strategies honing in on immune cell targeting within the TME. 

Bearing this in mind, the question of whether directing therapeutic intervention toward the dysregulated AT microenvironment could be efficacious arises (Table 1). 

Could excess weight, a primary contributor to this deregulated microenvironment, be easily overcome with weight loss? What could be the benefits of such intervention regarding prevention, outcomes, or cancer survivorship? The answer is unclear, as some studies suggest that this measure would not be enough to effectively resolve the profound changes and chronic inflammation induced by obesity [96,97]. Some preclinical studies on lung cancer have demonstrated that caloric restriction significantly improves immune surveillance against cancer by effectively reducing the number of immune-suppressive Treg cells in the TME [98]. In addition to reducing caloric intake, the pace of caloric intake and the daily intake profile could also matter. For instance, the fasting-mimicking diet significantly boosts CD8+ cytotoxic T-cell infiltration in breast and melanoma tumors, thereby strengthening immune surveillance and slowing down progression [91].

Studies have indicated that adopting specific diets, such as the Mediterranean diet, can improve metabolic syndrome indicators for obese individuals. This may include a reduction in inflammatory factors in the bloodstream and an increase in insulin sensitivity. Furthermore, research has demonstrated that a low-carbohydrate diet can be more effective than a low-fat diet in promoting weight loss and improving lipid profiles and insulin sensitivity. Nevertheless, it remains uncertain whether making these dietary adjustments can have a beneficial impact on cancer outcomes [99,100,101].

In addition to changes in diet, engaging in physical activity is a crucial factor. Moderate-to-intense exercise was found to lower systemic biomarkers of metabolic syndrome and increase adiponectin in overweight or obese breast cancer survivors [102].

Still, in the context of weight loss, it has been suggested that sympathomimetic agents that suppress appetite (phentermine or lorcaserin) or activate thermogenesis (such as mirabegron) might help reduce weight, WAT inflammation, and cancer risk [103].

Bariatric surgeries, including gastric bypass and banding, effectively boost weight loss, improve metabolic syndrome, and regularize the AT microenvironment. In obese patients, surgery promotes the downregulation of pro-inflammatory genes and upregulation of anti-inflammatory genes while significantly reducing the number of CLS in subcutaneous WAT [104]. In its retrospective study, Adams et al. [105] documented a reduction in all-cause mortality of 40% in obese patients who had undergone gastric bypass and a reduction in specific-cause mortality resulting from cancer (60%).

The modulation mechanism of the AT microenvironment with weight loss remains unclear. CLS formation was described in patients who underwent acute weight loss, suggesting an inflammatory remodeling of the AT, but this inflammatory response reflects the physiological behavior of a healthy AT expansion. Transcriptional changes in subcutaneous WAT and reduced levels of systemic biomarkers of metabolic syndrome were also described in the long-term follow-up of weight loss. This suggests that the formation of new CLS could be an essential mediator during rapid body-weight loss to tissue homeostasis. How these effects influence cancer remains unknown [106,107].

In addition to weight loss, directing the intervention to the metabolic syndrome could be relevant. Antidiabetic medications are currently under investigation for their potential in treating obesity. Metformin, for instance, induces not only a modest weight loss but also reduces the elevated levels of insulin associated with an inflamed AT [108]. Gliptins are another class of antidiabetic agents blocking dipeptidyl peptidase 4 (DPP4). One study demonstrated that the inflammatory pathways associated with obesity are regulated by liver-derived DPP4, so targeting DPP4 expression could be sufficient to prevent WAT inflammation and insulin resistance [109].

Ongoing investigations are being conducted to determine how lifestyle modifications can enhance cancer outcomes [110] (Table 1).

## 7. Conclusions

This review aimed to provide insight into the current understanding of the role of AT microenvironment in OC. The disruption of this microenvironment and CAAs are vital contributors to the processes of tumorigenesis and metastasis. CAAs may hold great promise in the development of treatments that can effectively reduce the impact of obesity on tumors.

Additional studies investigating the potential interaction of adipocytes with other cell types in the TME are warranted to determine if alterations in other cell types influence the biology of the tumor.

The correlation between cancer and disrupted AT is unquestionably significant. Further investigation into the interaction between adipocytes and OC is vital, as it can greatly impact adipocyte function. Therefore, it is crucial to prioritize translational research efforts to uncover the root causes of these associations and ultimately improve health outcomes for this rapidly growing demographic.

## Figures and Tables

**Table 1:** Clinical studies regarding cancer and the adipose tissue.

Concluded Preclinical Studies	Intervention		Results
Metformin limits the adipocyte tumor-promoting effect on OC [88]	Effect of metformin on adipocyte conditioned media and growth of ovarian tumor cells		Inhibition of adipocyte mediated OC tumorigenesis.
Epigenetic Targeting Of Adipocytes Inhibits High-grade Serous OC Cell Migration And Invasion [89]	Effect of treatment of adipocytes with guadecitabine in OC cells		Decrease in HGSOC migration and invasion.
Host obesity alters the ovarian tumor immune microenvironment and impacts response to standard of care chemotherapy [90]	Effect of a HFD vs LFD on response to standard of care chemotherapy on OC cell line and to assess obesity-associated changes in the TME		Significantly diminished response in HFD mice vs. LFD controls.
Fasting-Mimicking Diet Reduces HO-1 to Promote T Cell-Mediated Tumor Cytotoxicity [91]	Effect of combination of chemotherapy and a FMD on lymphoid progenitor cells and cytotoxic CD8(+) TILs		Enhancement of T-cell-dependent targeted killing of cancer cells.
**Randomized Clinical Trials**	**NCT Trial No.**	**Phase**	**Intervention**
Breast Cancer Weight Loss (BWEL) study [92]	NCT02750826	Phase III	Effect of telephone-based weight loss coaching in adjuvant treatment of BC.
Low Fat Versus Protein Sparing Diet for Weight Loss & Impact on Biomarkers Associated With Breast Cancer Risk (LEAF) [93]	NCT01559194	Phase III	Effect of LCD vs. LFD to determine the impact of these dietary patterns.
An Endometrial Cancer Chemoprevention Study of Metformin Versus No Treatment in Women with a BMI >/= 30 kg⋅m^2^ and Hyperinsulinemia [94]	NCT01697566	Phase III	Effect of metformin on the endometrium.
Diet and Physical Activity Change or Usual Care in Improving Progression-Free Survival in Patients With Previously Treated Stage II, III, or IV Ovarian, Fallopian Tube, or Primary Peritoneal Cancer [95]	NCT00719303	Phase III	Effect of a lifestyle intervention on PFS ovarian, fallopian tube or primary peritoneal cancer.

BC—breast cancer; BMI—body mass index; FMD—fasting-mimicking diet; HFD—high-fat diet; HGSOC—high-grade serous ovarian carcinoma; HO-1—heme oxygenase 1; LCD—low-carbohydrate diet; LFD—low-fat diet; OC—ovarian cancer; PFS—progression-free survival; TILs—tumor-infiltrating lymphocytes.

## Data Availability

No new data were created or analyzed in this study. Data sharing is not applicable to this article.
